# Recognition of Dual or Multiple Pathology in Skin Biopsies from Patients with HIV/AIDS

**DOI:** 10.4061/2011/398546

**Published:** 2011-06-22

**Authors:** Wayne Grayson

**Affiliations:** ^1^Drs Du Buisson, Kramer, Swart, Bouwer Inc., (AMPATH National Laboratories), Johannesburg 2092, South Africa; ^2^Division of Anatomical Pathology, School of Pathology, Faculty of Health Sciences, University of the Witwatersrand, Johannesburg 2193, South Africa

## Abstract

A large percentage of patients with HIV/AIDS will develop dermatological complications. Consequently, all practising clinicians and pathologists in regions with a high prevalence of HIV/AIDS must be familiar with the diverse cutaneous manifestations of the disease. This paper highlights the fact that biopsy material in this clinical context may occasionally reveal more than one pathological process. The potential spectrum includes two or more infections in a single skin biopsy (e.g., herpes simplex and cytomegalovirus infection), neoplastic lesions containing infective organisms (Kaposi sarcoma (KS) and cryptococcosis), dermatoses in association with neoplastic lesions (e.g., KS and interface dermatitis), or more than one dermatosis in a given specimen (e.g., papulopruritic eruption and nodular prurigo). Rare biopsies may even demonstrate triple pathology. The importance of careful examination of skin biopsies in this clinical context is emphasised. Failure to recognise an undiagnosed concomitant opportunistic infective pathogen could have potentially disastrous consequences for the patient.

## 1. Introduction

Dermatological disorders are common in patients with human immunodeficiency virus (HIV) infection and the acquired immunodeficiency syndrome (AIDS). It is said that more than 90% of HIV+ patients will develop cutaneous complications during the course of their illness, and in a significant proportion of these individuals, skin lesions may be a presenting symptom or sign of previously undiagnosed HIV infection [1, 2]. The spectrum of HIV-related skin disease includes a wide range of noninfective dermatoses, infective conditions, adverse drug reactions, and neoplastic proliferations [1]. It is not uncommon for this group of patients to manifest with more than one concurrent integumentary condition. Consequently, any given skin biopsy from an individual with cutaneous lesions in the presence of underlying HIV/AIDS could potentially reveal more than one pathological process. In severely immunosuppressed patients who have not received highly active antiretroviral therapy (HAART), subtle features of a coincidental potentially lethal opportunistic pathogen (e.g., cryptococcosis) can easily be overlooked in a specimen predominated by the primary pathological lesion (e.g., Kaposi sarcoma (KS)) [1, 3, 4]. Although dual or multiple pathological processes are seldom encountered in single skin biopsies in everyday practise, it is always wise to maintain a high index of suspicion when examining specimens from HIV/AIDS patients. 

The main objective of this paper is to draw the surgical pathologist's attention to the potential spectrum of dual or multiple pathology, using personally encountered case examples to illustrate this uncommon phenomenon.

## 2. Clinicopathological Spectrum

### 2.1. Overview

Surprisingly little has been written on the subject, with single case reports comprising most of the rare examples cited in the literature. Consequently, it is difficult to ascertain what proportion of skin biopsies from patients with HIV/AIDS might harbour dual or multiple pathological lesions. Based on the author's experience, it would seem that a combination of lesions is probably encountered in less than 2% of cutaneous histological samples obtained from this group of patients. In a vast majority of documented cases, the detection of combined pathology has been a reflection of marked underlying immunosuppression. Some more recently reported examples, however, have been a manifestation of the immune reconstitution inflammatory syndrome (IRIS) [3–5]. Although a detailed account of the cutaneous manifestations of IRIS is beyond the remit of this paper, many of the viral, fungal and bacterial infections alluded to below, and even neoplasms such as KS, may be a reflection of IRIS [3]. Satisfactory clinicopathological correlation is thus facilitated by knowledge of the patient's CD4 T-cell count and an indication of whether or not HAART has been implemented. 

The wide spectrum of potential colesional skin biopsy pathology is outlined in Table 1. This spectrum encompasses dual noninfective dermatoses, dual or multiple infections, neoplasia (KS) in association with infection, neoplasia (KS) with an associated dermatosis, mixed infective and inflammatory dermatoses, dual neoplastic conditions, and multiple pathologies. Although this latter group is uncommon, a high index of suspicion should always be maintained [1]. In most cases, the detection of more than one pathological lesion in a single biopsy is fortuitous and unexpected. On other occasions, however, a detailed history might reveal that the patient has clinical evidence of more than one type of skin lesion. Some dermatologists may therefore elect to intentionally sample overlapping lesions in a single biopsy [1].

### 2.2. Dual Noninfective Dermatoses

**Figure 1:**
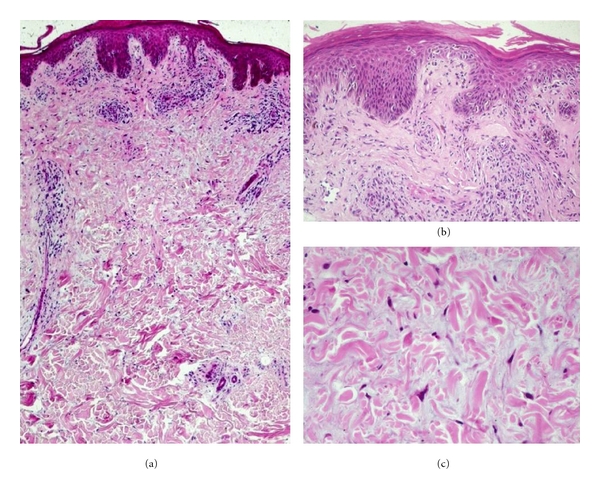
Papular mucinosis of HIV/AIDS. A mild superficial dermal mononuclear inflammatory infiltrate is associated with subacute eczematous changes in the overlying epidermis (a, b), while the deeper dermis shows separation of collagen bundles by interstitial mucin (c).

Since HIV/AIDS may be associated with a diverse array of noninfective dermatological disorders, and patients may often have more than one skin disease at presentation, it is somewhat surprising that skin biopsy specimens seldom reveal dual pathology of a noninfective nature. Two conditions, however, are known to sometimes demonstrate dual pathology. The first of these is pruritic papular eruption of HIV (papulopruritic eruption), where the longstanding pruritis and excoriation may be complicated by superimposed changes of nodular prurigo [6]. The second condition is papular mucinosis of HIV/AIDS, where intradermal mucin deposition is accompanied by concomitant epidermal changes of an eczematous dermatitis, as illustrated in Figure 1 [6–8].

### 2.3. Dual Infections

**Figure 2:**
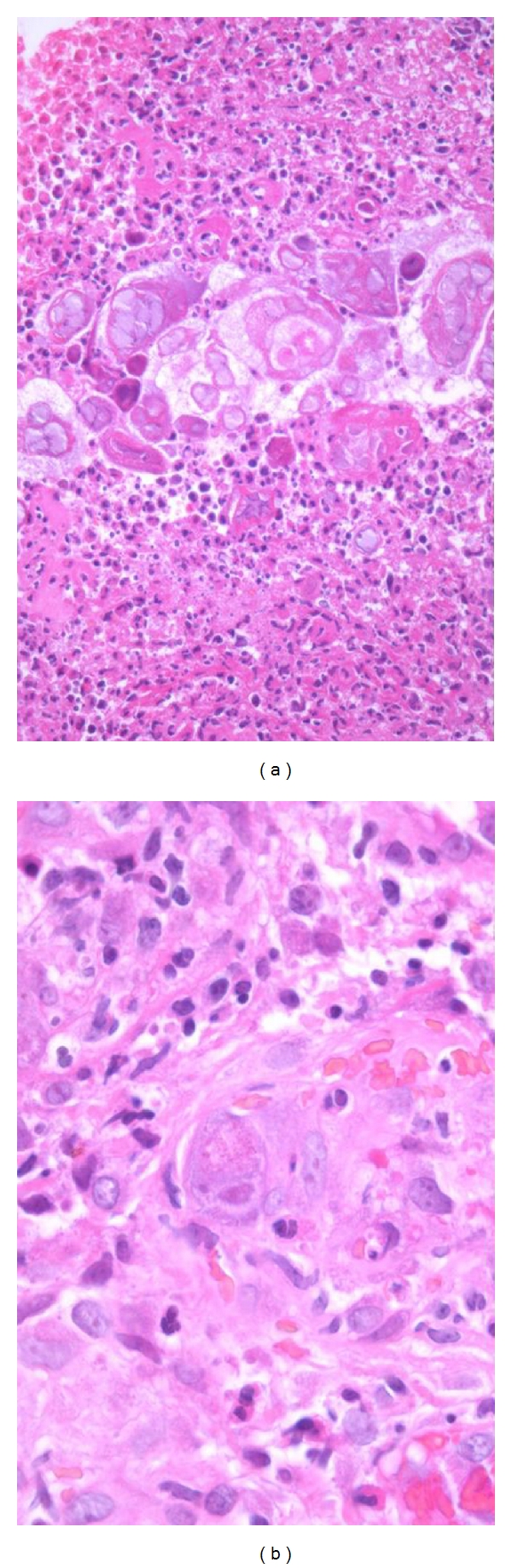
Biopsy of a vulval ulcer in a female patient with AIDS. (a) The ulcerated surface epithelium harbours many intranuclear herpes simplex virus inclusions. (b) Concomitant cytomegalovirus infection of an endothelial cell in the inflamed underlying dermis.

The increased susceptibility to infection by a wide range of opportunistic pathogens is a hallmark of HIV/AIDS. It is, therefore, essential that histological specimens in this clinical context are always examined carefully to ensure that a second (or perhaps even a third) infective organism is not overlooked [1]. The most frequently encountered copathogen is cytomegalovirus (CMV). CMV is often identified in the dermis in biopsies obtained from genital or perineal ulcers caused by herpes simplex virus (Figure 2), and associated vasculitis is not uncommon [1, 9–11].

**Figure 3:**
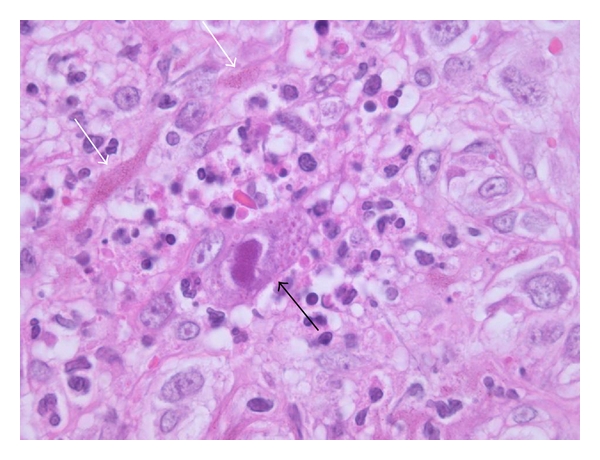
Bacillary angiomatosis with incidental cytomegalovirus (CMV) infection (black arrow). Granular colonies of extracellular *Bartonella *bacilli (white arrows) are present amid the background endothelial cell proliferation and infiltrate of polymorphonuclear leucocytes.

**Figure 4:**
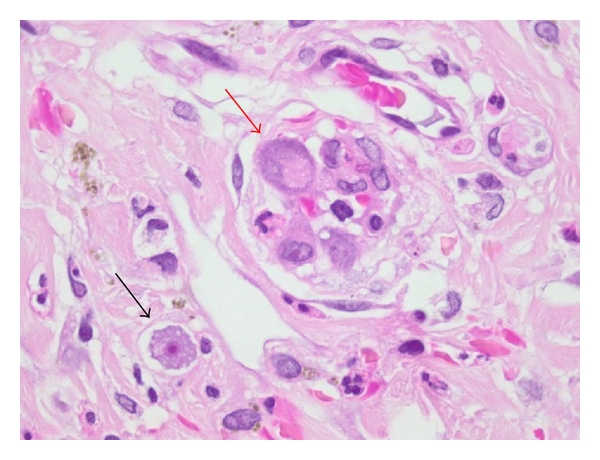
Cutaneous *Acanthamoeba* infection (black arrow) with concomitant cytomegalovirus (CMV) infection (red arrow) in biopsy from a patient with advanced AIDS and widespread cutaneous ulceration.

CMV infection has also been documented in association with bacillary angiomatosis (BA) (Figure 3) and mucormycosis [12, 13]. The author has also encountered a case of widespread cutaneous ulceration due to vasculitis in the presence of acanthamoebiasis and concomitant CMV infection (Figure 4). Although skin lesions due to infection with *Acanthamoeba *spp. may occur in patients with AIDS, to date there have been no additional examples with CMV coinfection recorded in the literature [14–17]. 

Cutaneous infection with *Pneumocystis jiroveci *(formerly *P. carinii*) is a rare complication of HIV/AIDS. There have, however, been single case reports of coinfection with other organisms, namely, botryomycosis due to concomitant *Staphylococcus aureus *infection, and skin lesions harboring both *Pneumocystis *organisms and *Cryptococcus neoformans* yeasts [18, 19]. BA has been reported in association with *Mycobacterium avium-intracellulare *(MAI) infection [5, 20]. Cases with lesions containing three separate infective organisms are discussed in Section 2.8. 

### 2.4. Dual Infective and Noninfective Dermatoses

**Figure 5:**
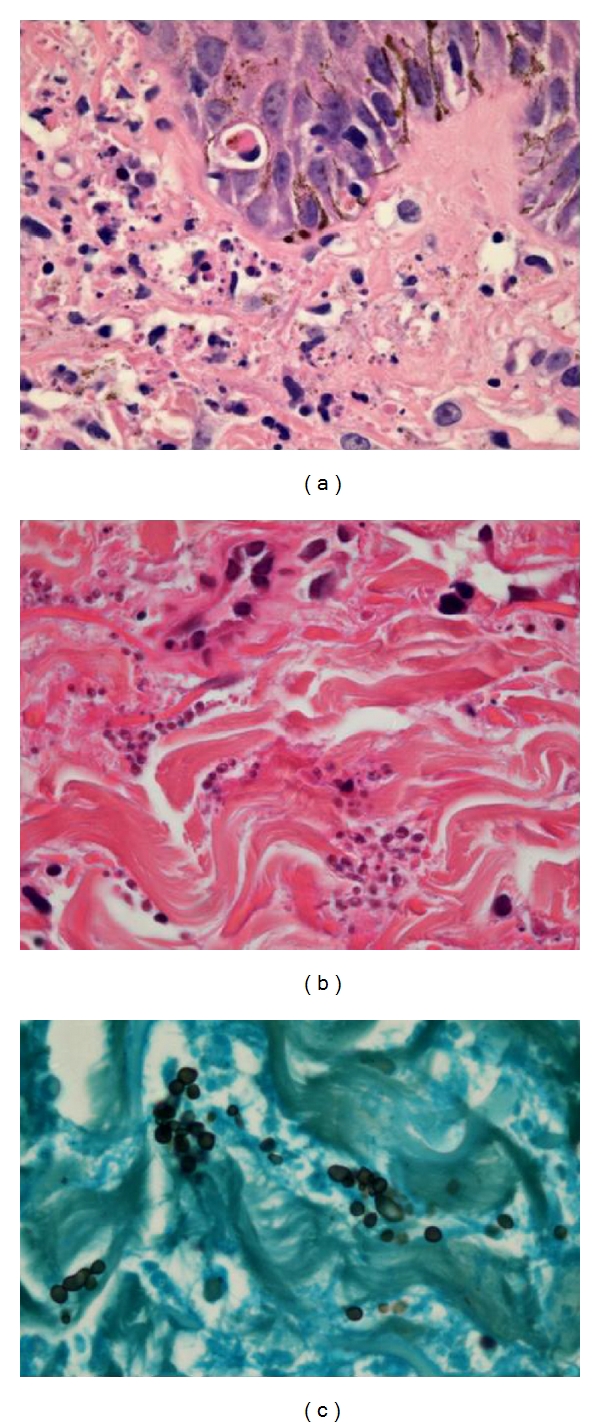
Skin biopsy from a patient with AIDS-associated histoplasmosis and concomitant erythema multiforme. A lichenoid reaction is visible in relation to the dermoepidermal interface (a), while the dermis contains conspicuous numbers of *Histoplasma capsulatum* yeasts (b), whose presence is highlighted with the aid of a Grocott stain (c).

**Figure 6:**
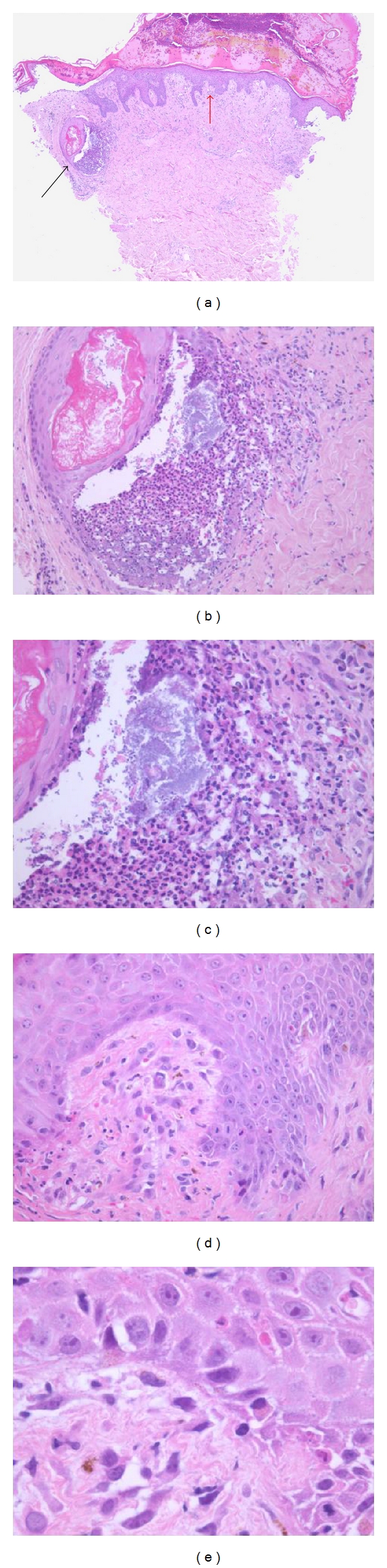
(a) Suppurative folliculitis (black arrow) associated with interface dermatitis (red arrow), the latter ascribed to recently introduced HAART. There is a florid folliculocentric neutrophilic infiltrate (b), with the lumen of the partially disrupted follicle containing both *Staphylococcus *organisms and *Malassezia *yeasts (c). Drug-induced pauci-inflammatory interface dermatitis is observed in the neighbouring epidermis (d, e). (By courtesy of Dr. J. Rigby, National Health Laboratory Service and the University of the Witwatersrand, Johannesburg, South Africa.)

This phenomenon has, to the best of one's knowledge, not been addressed in the literature. Vacuolar interface dermatitis is a reaction pattern with a number of potential causes in patients with HIV/AIDS. Causes are diverse, including the acute exanthem of HIV infection, a morbilliform drug rash, erythema multiforme, and so-called AIDS interface dermatitis [1]. Doubt has been expressed regarding the validity of the latter as a distinct clinicopathological entity, as a significant proportion of patients whose biopsies revealed interface dermatitis had one or more opportunistic infections and/or had been receiving one or more drugs at the time of diagnosis [1, 6, 21, 22]. There are nevertheless occasions in which interface dermatitis is detected coincidentally in a biopsy performed for another reason (e.g., confirmation of KS) and in which there is no significant drug history (see below) [1, 6]. The author has encountered cases where careful clinical correlation has elucidated the cause of this reaction pattern when a skin biopsy has been performed for confirmation of another condition. Anecdotal examples include histoplasmosis with erythema multiforme (Figure 5), and folliculitis in association with an adverse drug reaction to HAART (Figure 6).

### 2.5. Neoplasia in Association with Infection

**Figure 7:**
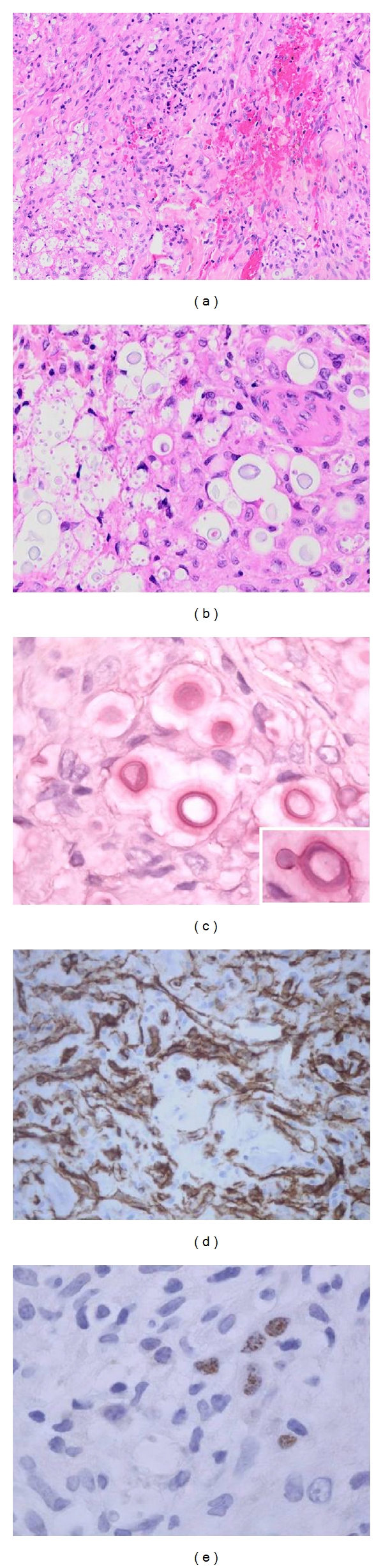
(a) AIDS-associated Kaposi sarcoma with concomitant cutaneous cryptococcosis. *Cryptococcus neoformans *yeasts (including capsule deficient forms) are present amid the background spindle cell proliferation (b), with the mucicarmine preparation (c) highlighting the mucoid capsule around individual yeasts; narrow-based budding is present (inset). The KS lesional cells demonstrate immunoreactivity for both CD31 (d) and human herpes virus type 8 (HHV-8) (e).

KS skin lesions may rarely contain coincidental infective organisms. Failure to recognise the opportunistic pathogen, whose presence is often masked by the more obvious background spindle cell proliferation, could have grave prognostic implications. To date there have been only nine recorded cases of AIDS-associated KS with colesional cryptococcosis, one of whom had oral lesions exclusively [4, 23–27]. The author has encountered three additional examples of KS with incidental *Cryptococcus neoformans *infection, including the case illustrated in Figure 7. 

*Histoplasma capsulatum *coexistent with KS in a single lesion has also been reported [28]. In this context, *H. capsulatum *yeasts should be distinguished from infection with capsule deficient forms of *C. neoformans *occurring in association with KS [3, 4]. There are two documented cases of KS with apparent intralesional *Candida *organisms. The latter were identified on ultrastructural examination [29]. Although there are rare reports of patients with concurrent KS and BA, these cases seem to have presented with separate lesions, showing no apparent microscopic evidence of BA in the KS histological specimens and *vice versa* [30–32]. 

**Figure 8:**
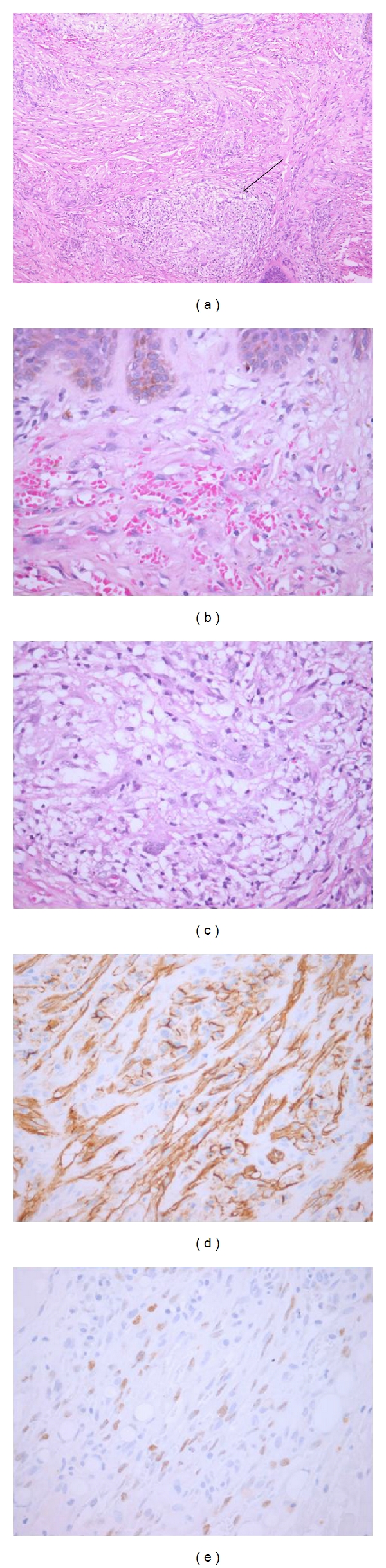
(a) Facial Kaposi sarcoma (KS) in an adult man undergoing treatment for pulmonary tuberculosis. A noncaseating granuloma is present within the KS lesion in the lower half of the field (arrow). (b) Detail of the vasoformative KS proliferation. (c) Higher magnification of the tuberculous granuloma. Although acid-fast bacilli could not be demonstrated on Ziehl-Neelsen staining, mycobacterial DNA was detected by PCR. (d) CD31 immunostain highlighting the background KS. (e) The KS lesional cells were also immunoreactive for HHV-8.

MAI infection has uncommonly been documented in association with KS, including one patient who had a facial non-Hodgkin lymphoma of immunoblastic type containing acid-fast organisms, and another who had concomitant colesional cryptococcosis [26, 33–35]. More recently, *M. tuberculosis *has also been detected in KS cutaneous lesional tissue (Figure 8), where its presence may serve as a clue to underlying HIV infection, systemic tuberculosis, noncompliance to antituberculous medication, or multidrug resistant tuberculosis [36]. Although these tuberculoid granulomas are recognised with relative ease in involved KS lesions, mycobacterial spindle cell tumours concomitant with KS may pose a particular diagnostic challenge [3]. This latter phenomenon has been described in the lymph nodes of HIV+ patients but has not, to the best of one's knowledge, been documented in the skin [37]. 

Not unexpectedly, KS may occasionally harbour CMV inclusions, including KS involving the oral cavity [3, 38, 39]. Although the clinical significance thereof is the subject of debate, the identification of CMV inclusions in KS may serve as a sentinel for more serious systemic CMV disease such as retinitis or pneumonitis [3, 38]. The fortuitous detection of molluscum contagiosum in KS has also been reported; the presence of both lesions in a single histological specimen may be the first clue to underlying HIV infection/AIDS [3, 40].

### 2.6. Neoplasia in Association with Noninfective Dermatoses

Since patients with HIV/AIDS often present with cutaneous lesions caused by more than one disease, it is not entirely unexpected that biopsies performed for histological confirmation of KS may sometimes show evidence of a concomitant noninfective dermatosis. Although uncommon, it has been the author's experience that incidental interface dermatitis is encountered occasionally in the epidermis overlying a KS lesion [6, 41]. While some examples are almost certainly attributable to a concurrent adverse drug reaction, some cases have no significant pharmacological history and could perhaps represent examples of so-called “AIDS interface dermatitis” [1, 6, 21, 22]. 

**Figure 9:**
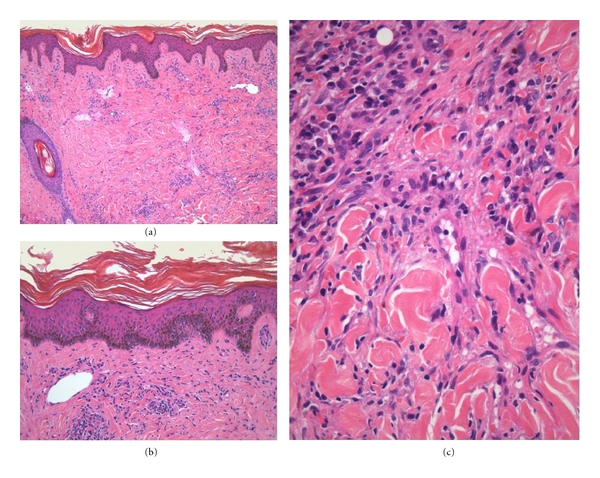
Skin biopsy from a patient with HIV-associated acquired ichthyosis and Kaposi sarcoma. The epidermis displays prominent hyperkeratosis (a, b), while the underlying dermis is expanded by a typical plaque-stage Kaposi sarcoma proliferation (c).

There is a well-documented association between AIDS and acquired ichthyosis, including patients who have presented with concomitant KS [42–44]. Biopsies from such patients may therefore reveal histological features of both conditions, as depicted in Figure 9 [41]. The latter biopsy was intentionally obtained from an area of ichthyosis overlying a suspected dermal KS lesion on the lower extremity.

### 2.7. Dual Neoplastic Lesions

**Figure 10:**
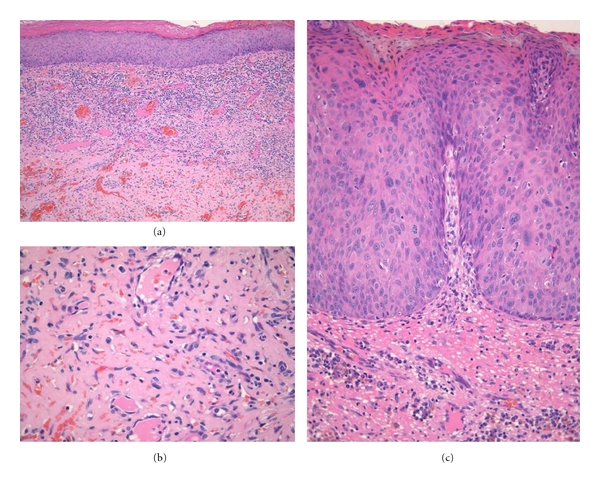
(a) Penile squamous cell carcinoma *in situ *in association with genital Kaposi sarcoma. (b) Detail of the Kaposi sarcoma proliferation replacing the subepithelial stroma. (c) Severe dysplasia involving the full thickness of the overlying surface epithelium.

AIDS carries an increased risk of oral and anogenital neoplasia [6, 45, 46]. Rarely, biopsies of KS genital lesions may reveal coincidental squamous cell carcinoma *in situ*, as illustrated in Figure 10 [41].

### 2.8. Multiple Pathology

**Figure 11:**
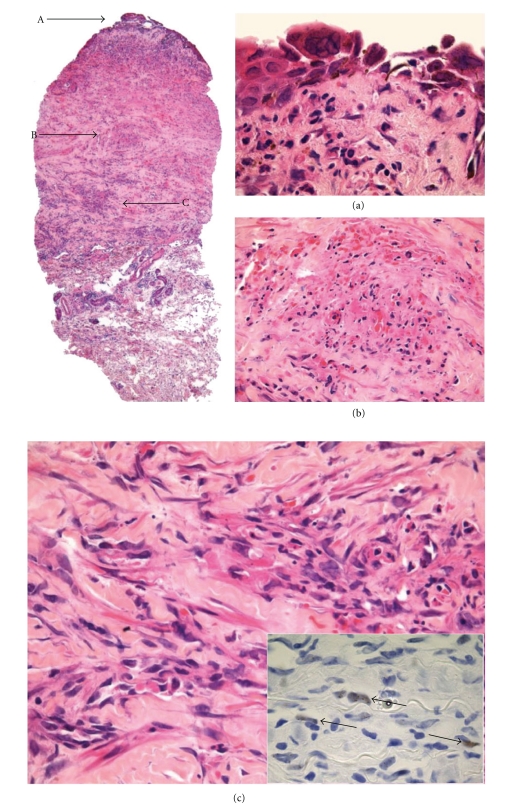
Skin biopsy demonstrating multiple HIV/AIDS-related pathology, including a superficial vesicle due to varicella-zoster virus infection (a), leucocytoclastic vasculitis in the mid- to upper dermis (b), and incidental Kaposi sarcoma in the deeper dermis (c), the latter confirmed by immunohistochemical staining for HHV-8 (inset). (Reproduced from [1] with permission from BMJ Publishing Group Ltd.).

Although rare, single histological specimens may occasionally reveal more than two pathological lesions. This rare phenomenon has been documented in single case reports. Most comprise either three separate infections, or two infections in association with KS. Recorded examples include the following:

- BA, MAI, and CMV coinfection [47, 48],
- Concurrent CMV, MAI, and *M. tuberculosis* infection [49],
- Infection with *S. aureus*, CMV, and *Mycobacterium *spp. [50],
- MAI, KS, & *Cryptococcus* infection [26], and
- varicella-zoster virus (VZV) infection, leucocytoclastic vasculitis, and KS (Figure 11) [1].

The patient whose biopsy is depicted in Figure 11 had purple-red KS lesions and an associated vesicular eruption. The sample was intentionally obtained from a somewhat haemorrhagic lesion on the trunk, where a vesicle was observed on the surface of a clinical KS lesion.

## 3. Conclusion

Colesional pathology may easily be overlooked in skin biopsies from patients with HIV/AIDS, especially when the primary pathological lesion dominates the histological picture. It is wise for the surgical pathologist to always consider dual or multiple pathology when examining biopsy material from this group of patients. Rigorous adherence to this principle when studying the routine haematoxylin and eosin (H&E) stained sections will usually facilitate diagnosis or at least raise suspicion for a second or perhaps even a third pathological process. Since it is neither practical nor cost-effective to perform a panel of histochemical and/or immunohistochemical stains for potential identification of infectious organisms on each and every biopsy, relevant stains should be reserved for confirmation of one's suspicion based on the H&E findings. If necessary, additional biopsies should be recommended for appropriate microbiological studies, such as fungal culture. A clinical history of polymorphous skin lesions should alert the pathologist to the potential for dual or multiple pathology in a given skin biopsy. 

CMV remains the most frequently encountered coincidental pathogen, and whilst its precise pathological significance is often uncertain, one should remain cognisant that the presence of CMV inclusions in a skin biopsy may serve as a sentinel for more serious systemic involvement [3]. The detection of tuberculous granulomas or *Cryptococcus *organisms in KS has similar implications [3, 4, 36]. Although the patients whose biopsies are illustrated herein were largely HAART-naïve, awareness that colesional pathology may be a rare manifestation of IRIS is appropriate in the era of HAART. A potentially interesting collaborative research opportunity may reside in a detailed clinicopathological analysis of a series of such cases.

## Figures and Tables

**Table 1:** Overview of the potential spectrum of dual or multiple pathology in skin biopsies from patients with HIV/AIDS.

Pathology	Reference(s)
Dual noninfective dermatoses	
PPE & nodular prurigo	[1, 6]
Papular mucinosis & eczematous dermatitis	[1, 6–8]

Dual infections	
HSV & CMV	[9–11]
BA & CMV	[12]
Mucormycosis & CMV	[13]
Acanthamoebiasis & CMV	U
Pneumocystosis & *S. aureus * (botryomycosis)	[18]
Pneumocystosis & cryptococcosis	[19]
MAI & BA	[5, 20]

Dual infective and noninfective dermatoses	
Histoplasmosis & erythema multiforme	U
Folliculitis & interface dermatitis	U

Neoplasia in association with infection	
KS & cryptococcosis	[3, 4, 23–25]
KS & *Histoplasma capsulatum *infection	[28]
KS & *Candida *infection	[29]
KS & MAI infection	[33–35]
KS & Tb	[3, 37]
KS & CMV	[3, 38]
KS & molluscum contagiosum	[3, 40]
NHL & MAI infection	[35]
(KS & BA*)	[30, 32]

Neoplasia in association with noninfective dermatoses	
KS & incidental interface dermatitis	[1, 6]
KS & acquired ichthyosis	[41]

Dual neoplastic lesions	
KS & penile squamous cell carcinoma *in situ *	[41]

Multiple pathology	
MAI infection, KS, & cryptococcosis	[26]
BA, MAI, & CMV	[47, 48]
MAI & CMV & Tb	[49]
*S. aureus*, CMV, & *Mycobacterium *spp.	[50]
VZV infection, LCV, & KS	[1]

PPE: pruritic papular eruption of HIV; HSV: herpes simplex virus; CMV: cytomegalovirus; BA: bacillary angiomatosis; U: hitherto unreported; MAI: *Mycobacterium avium-intracellulare *complex; *S. aureus*: *Staphylococcus aureus*; Tb: *Mycobacterium tuberculosis*; KS: Kaposi sarcoma; NHL: non-Hodgkin lymphoma; VZV: varicella-zoster virus; LCV: leucocytoclastic vasculitis.

*Recorded concurrently in the same patient, but not in the same histological specimen.
